# Lactate and lactylation in macrophage polarization of sepsis

**DOI:** 10.3389/fcimb.2025.1662444

**Published:** 2026-01-12

**Authors:** Jian Sun, Min Zhang, Li Zhang, Xinyue Zhang, Jingxiao Zhang

**Affiliations:** Department of Emergency Critical Care Medicine, The Second Hospital of Jilin University, Changchun, China

**Keywords:** lactate, lactylation, macrophage polarization, metabolic reprogramming, sepsis

## Abstract

Sepsis, which is characterized by potentially fatal multiple organ dysfunction, is caused by an abnormal host response to a major infection. During sepsis, the pathogen stimulates the host to activate resistance mechanisms that enhance immune cells’ oxygen consumption in inflammatory tissues and cells, and promote aerobic glycolysis. Lactate generated by aerobic glycolysis is an essential substrate for the tricarboxylic acid cycle and for post-translational modifications via histone lactylation and epigenetic regulation. It also serves as a signaling molecule that modulates macrophage polarization between pro- and anti-inflammatory phenotypes in response to inflammatory and metabolic signals in their local environment. The roles of lactate and lactylation modifications in cancer cell proliferation and invasion have been well studied and are now potential therapeutic targets for various malignancies. However, the roles of lactate and lactylation modification in sepsis remain unclear. This review focuses on lactate’s regulatory mechanism and lactylation modification during macrophage polarization in sepsis, and investigates whether this regulation could be a potential therapeutic target for sepsis.

## 1 Introduction

Sepsis is a result of an aberrant host response to serious infection, characterized by life-threatening multiple organ dysfunction (Singer et al., 2016; Backer et al., 2024). It has been estimated that sepsis affects about 50 million people globally per annum and remains a major cause of death, increasing the global health burden (Fleischmann-Struzek et al., 2020; Rudd et al., 2020). The host initiates a vigorous anti-infection response to the pathogen in sepsis (Binnie et al., 2020). Resistance and tolerance mechanisms are two evolutionarily conserved defense systems that are essential for both efficient immune responses and host survival in sepsis (Willmann and Moita, 2024). While disease tolerance systems improve host fitness by reducing and repairing tissue damage (Martins et al., 2019) caused by both the pathogen and excessive inflammation without affecting pathogen load, resistance mechanisms attempt to lower the pathogen burden directly (Soares et al., 2017). Macrophages, as key mediators of immune activity in sepsis, demonstrate functional flexibility by switching between pro- and anti-inflammatory phenotypes based on the inflammatory and metabolic signals in their local environment. In addition to their antimicrobial roles in resistance mechanisms against infections, macrophages also maintain local and systemic homeostasis (Wculek et al., 2022), and their metabolic reprogramming plays a crucial part in developing disease tolerance (Ip et al., 2017).

Lactate serve as a significant biomarker for sepsis, exhibiting a favorable correlation with death and morbidity in cases of septic shock or sepsis (Mao et al., 2022; Schneider et al., 2022). It has been observed that during sepsis, lactate is produced by aerobic glycolysis or bacterial stimulation, which modulates macrophage polarization and the levels of certain genes *via* specific signaling pathways (Dichtl et al., 2021; Llibre et al., 2025). Moreover, lactate is present at the junction of oxidative and glycolytic metabolisms, serving as a vital energy intermediate. In 2014, Colegio et al. discovered that lactate can be absorbed by tumor-associated macrophages through the monocarboxylate transporter 1 (MCT1) in a melanoma model, subsequently directing macrophages towards a pro-tumorigenic phenotype (Colegio et al., 2014). Lactate has since been regarded as a significant substrate in the tricarboxylic acid (TCA) cycle (Hui et al., 2017) and post-translational modifications (PTMs) through histone lactylation and epigenetic modulation (Zhang et al., 2019). It also functions as a signaling molecule that modulates both innate (Colegio et al., 2014) and adaptive immune responses, as required (Pucino et al., 2019). The roles of lactate and lactylation modification in cancer cell proliferation and invasion have been well studied and become potential therapeutic targets for various malignancies (Colegio et al., 2014; Zhang et al., 2019). However, the roles of lactate and lactylation modification in sepsis remain unclear, this review focuses on lactate’s regulatory mechanism and lactylation modification in macrophage polarization during sepsis, and investigates whether the regulation would be a potential therapy target for sepsis.

## 2 Metabolic reprogramming of macrophages in sepsis

According to historical descriptions, sepsis progresses in two stages: an initial hyperinflammatory stage caused by an acute burst of pro-inflammatory mediators and metabolic deviation in response to injury or infection, and a later immunosuppressive stage distinguished by counter-regulatory mechanisms to limit and control the inflammatory process, facilitate repair mechanisms, and return to basal homeostasis (Van Wyngene et al., 2018; Willmann and Moita, 2024). Macrophages are very flexible and can polarize into several phenotypes in response to stimuli (Yu et al., 2017). LPS-stimulated macrophages, commonly referred to as M1, exhibit a decrease in oxidative phosphorylation (OXPHOS) and an increase in aerobic glycolysis (also known as the Warburg effect). Glycolysis is up-regulated, which aids macrophages in fully developing their pro-inflammatory phenotype (Semba et al., 2016; Lauterbach et al., 2019). On the other hand, macrophages treated with interleukin-4 (IL-4), also known as M2, display alternative activation markers, such as arginase 1 (Arg1), resistin-like molecule α (RELM-α), and PD-1 ligand 2 (PD-L2), and up-regulate OXPHOS (Huang et al., 2016).

**Figure 1:**
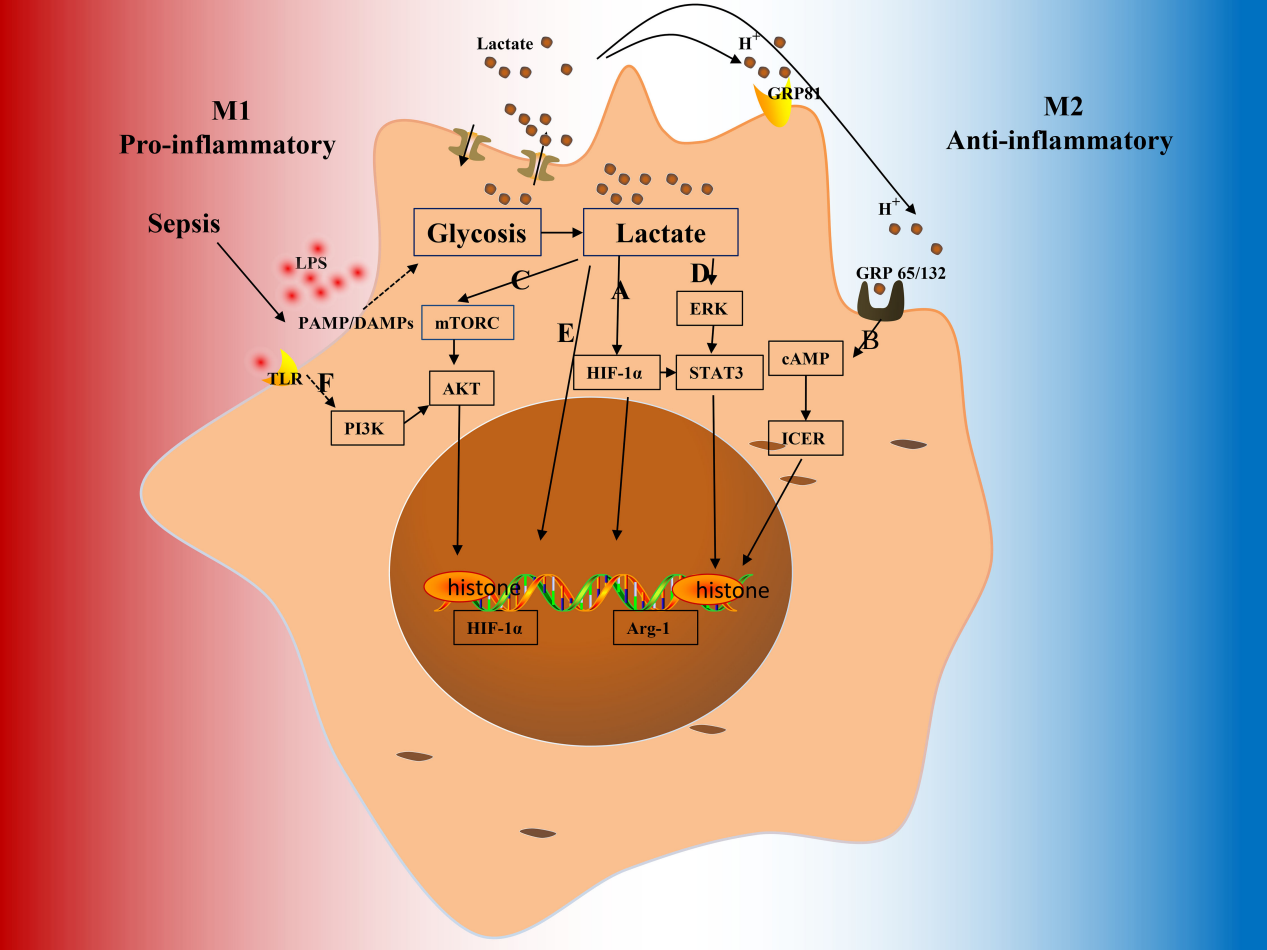
The pathway through which lactate induces M2 macrophage phenotype in sepsis. **(A)** Lactate increases the levels of VEGF and Arg-1 by stabilizing/activating HIF-1α. **(B)** Extracellular lactate promotes acidic TME, which activates proton-sensing GPR65/GPR132, mediating the expression of M2-like genes through the cAMP-ICER pathway. **(C)** Lactate activates the mTOR-AKT pathway, which modulates TAM to a proinvasive M2-like phenotype. **(D)** Lactate stimulates the ERK-STAT3 pathway through ERK phosphorylation, which then promotes pro-angiogenic and anti-inflammatory M2-like phenotypes. **(E)** Lactate induces histone lactylation at the H3K18 promoter site of VEGF, Arg-1, and various M2-like genes in TAM to increase their levels, thus mediating an M2-like phenotype in TAM. **(F)** TLR can activate PI3K-AKT, which promotes the transformation of macrophages into an anti-inflammatory repair phenotype *via* the PI3K-AKT-GSK3b-FOXO1 pathway. TME tumor mircro environment, mTOR mammalian target of rapamycin, GPR81 G-protein-coupled receptor 81, TLR4 toll-like receptor 4, HIF-1α hypoxia inducible factor-1α.

M1 macrophages show decreased OXPHOS through the TCA cycle, higher glucose absorption, an increased rate of glycolysis, and an up-regulated pentose phosphate pathway during the early stages of sepsis (Tannahill et al., 2013). This metabolic shift in highly glycolytic, activated “M1” macrophages has led to increased levels of the TCA cycle intermediate succinate, which has been shown to enhance the signal that stabilizes hypoxia-inducible factor-1α (HIF-1α) (**Figure 1**) (Koivunen et al., 2007; Tannahill et al., 2013). This process occurs when extra succinate is transferred from the mitochondria into the cytosol, where it might hinder the activity of the prolyl hydroxylase domain (PHD), stabilizing and activating HIF-1α. The proinflammatory cytokine IL-1β and other HIF-dependent genes, such as those encoding glycolysis-related enzymes, are subsequently positively regulated by HIF-1α (Tannahill et al., 2013).

Following the acute inflammatory phase of sepsis, the body uses a variety of counter-regulatory mechanisms to limit the inflammatory process, facilitate repair, and return to basal homeostasis as tissue damage increases, immune cells undergo metabolic reprogramming, the inflammatory microenvironment changes, and metabolic byproducts accumulate (Willmann and Moita, 2024). The establishment of disease tolerance is dependent mainly on macrophage metabolic reprogramming, potentially mediated by IL-10 (Ip et al., 2017). This cytokine inhibits glycolysis and glucose absorption while promoting OXPHOS. Furthermore, IL-10 stimulates the expression of DNA damage inducible transcript 4 (DDIT4), which inhibits the activity of the mammalian target of rapamycin (mTOR). This results in the mitophagy of malfunctioning mitochondria, which lowers ROS levels, inhibits NOD-like receptor thermal protein domain-associated protein 3 (NLRP3) inflammasome activation, and reduces IL-1 production, all of which can harm cells and tissues (Ip et al., 2017).

## 3 The influence of lactate-related signaling pathways on macrophage polarization in sepsis

### 3.1 Lactate changes in sepsis

Lactate is one of the primary metabolites of glycolysis in sepsis and plays a significant role in resistance and tolerance mechanisms (Willmann and Moita, 2024). Serum lactate levels serve as a significant biomarker for sepsis (Mao et al., 2022; Schneider et al., 2022). It is becoming increasingly clear that not only accelerated aerobic glycolysis flux but also reduced hepatic clearance contribute to hyperlactatemia (Suetrong and Walley, 2016). However, 24–72 hours post-tissue injury, they transition to anti-inflammatory phenotypes, enhancing mitochondrial metabolic pathways and suppressing glycolytic genes, facilitating tissue repair and restoring homeostasis (Varga et al., 2016). Furthermore, studies on immune metabolism have indicated that aerobic glycolysis induced immune cell activation and lactate generation may promote an immunosuppression of the local inflammation and innate immunity (Nolt et al., 2018; Lee et al., 2022). These metabolic changes have profound implications for immune control and organ function, modulating the pathogenesis of sepsis.

Relative to other immune modulatory metabolites, like itaconate, fumarate, and succinate, lactate’s signaling pathways and sensing are evolutionarily markedly developed (Zasłona and O’Neill, 2020). Lactate acts as an essential glycolytic precursor, a major source of energy for mitochondrial respiration, and a signaling molecule, by shuttling between inter- and intra-cellular compartments (Brooks, 2020). Its complex network comprises 2 transporter families: monocarboxylate transporter 1(MCT1), MCT4 (Felmlee et al., 2020) and 2 sodium-coupled MCTs (SLC5A8 and SLC5A12) (Ganapathy et al., 2008) as well as membrane receptors, G-protein-coupled receptor 81 (GPR81) and GPR132 (Chen et al., 2017; Khatib-Massalha et al., 2020). MCT1 and MCT4 have been comprehensively researched. MCT1 is markedly found in multiple tissues and facilitates the uptake of lactate, whereas MCT4 is primarily present in tissues with excess glycolysis and promotes lactate outflow (Sun et al., 2017; Ye et al., 2022). Each lactate molecule produced releases one proton (H^+^), proton and lactate concentration gradients, however, determine the direction of *in vivo* MCT-mediated lactate transport. Lactate shuttling is facilitated by a variety of bodily systems and compartments, including the interstitial space, circulation, and vasculature (Tasdogan et al., 2020).

### 3.2 The impact of lactate on macrophage polarization in sepsis

The literature suggests that lactate modulates macrophage metabolic reprogramming in inflammation, cancer, and pulmonary fibrosis (Cui et al., 2021; Wang N. et al., 2022). Furthermore, it inhibits pro-inflammatory M1 macrophages and promotes pro-angiogenic and anti-inflammatory M2 phenotype. These effects are facilitated by multiple processes, including histone PTM and signaling pathway changes (Ivashkiv, 2020): (1) pyruvate metabolizes lactate through lactate dehydrogenase (LDH), which in turn results in the stabilization of HIF-1α by inhibiting prolyl hydroxylase. HIF-1α increases tumor-associated macrophages’ vascular endothelial growth factor (VEGF)and Arg-1 levels via a mechanism independent of IL-4/IL-13, inducing the M2 phenotype. HIF-1α, however, does not raise the levels of Arg-1 or VEGF during M1 polarization, as it is expressed early and induces the transcription of glycolytic genes, rather than Arg-1 and VEGF (Colegio et al., 2014; Zhang et al., 2019) (**Figure 1A**). (2) Lactate increases immunological tolerance by activating the mTOR, which subsequently reduces HIF-1α’s lysosomal degradation, thus enhancing macrophage anti-inflammatory responses (Liu et al., 2023) (**Figure 1C**). (3) In macrophages, lipopolysaccharide (LPS) recognition by toll-like receptor 4 (TLR4) stimulates nuclear factor kappa-B (NF-kB), which mediates pro-inflammatory molecules and elevates lactate production by increasing aerobic glycolysis (Zanoni et al., 2011; Fitzgerald and Kagan, 2020). Irizarry-Caro RA et al. showed that B-cell adapter for PI3K (BCAP) is a novel TLR signaling adapter, which modulates macrophage differentiation into an M2 repair phenotype (Troutman et al., 2012). TLR stimulates PI3K-AKT through BCAP, and active AKT induces M2 transition of macrophage phenotype *via* the PI3K-AKT-GSK3b-FOXO1 pathway. Furthermore, AKT phosphorylation suppresses GSK3b and FOXO1, its downstream targets, alleviating inflammation. The accumulation of lactate promotes histone lactylation, which increases wound repair gene levels (**Figure 1F**). These data suggest that BCAP has an essential switch-like role in the M2 repair phenotype differentiation of macrophages and serves as a potential pharmacological target for treating disorders associated with the excessive activation of the macrophage proinflammatory phenotype (Irizarry-Caro et al., 2020). Lactate suppresses NLRP2 and TLR4 signaling during inflammation, disrupting the NF-κB-IL-1β-Casp1 axis through GPR81 and arrestin β 2 (ARRB2) (Hoque et al., 2014). The expression of lactate is markedly high in the brain, which alters during pathological conditions. In microglia, lactate can promote pro-inflammatory (Andersson et al., 2005) and anti-inflammatory (Zhang et al., 2023) effects, as well as increased (Liu et al., 2020) and decreased (Nicola et al., 2022) phagocytic capacity, potentially *via* GPR81 but not MCT1 (Nicola et al., 2022). (4) GPR132 and GPR65 are primarily found on macrophage surfaces and are stimulated by the acidic TME derived from extracellular conditions. Moreover, they induce an intracellular signaling cascade, stimulating the production of pro-angiogenic and anti-inflammatory genes in macrophages through the cAMP-ICER pathway, thus facilitating M2 phenotype differentiation of macrophages (Chen et al., 2017; Bohn et al., 2018) (**Figure 1B**).

### 3.3 The impact of ketone bodies on macrophage polarization in sepsis

Lactate and pyruvate, the byproducts of glycolysis, offer negative feedback inhibition of glucose elimination (Brooks, 2020). In particular, the ketone body β-hydroxybutyrate may inhibit histone deacetylase activity (Chisolm and Weinmann, 2018), with clear health consequences, particularly for people who are malnourished or fasting, who eat high-fat, ketogenic diets, or who have severe insulin insufficiency and are at risk for ketoacidosis. It is anticipated that β-hydroxybutylation of histones will neutralize the positive charge on lysine residues, creating a more open chromatin for gene transcription, much as histone acetylation (Xie et al., 2016). The discovery that β-hydroxybutyrate can suppress histone deacetylase activity remains debated (Bendridi et al., 2022). Lactate can also inhibit histone deacetylases activity (Latham et al., 2012), potentially altering gene expression and raising interesting considerations about the potential effects of acute or chronic hyperlactatemia on epigenetics. Acetyl-CoA and Malonyl-CoA are produced from lactate, which is also the primary mitochondrial substrate. Malonyl-CoA prevents the absorption of derivatives of free fatty acids (FFAs) in the mitochondria through carnitine palmitoyltransferase1 (CPT1), whereas acetyl-CoA inhibits β-ketothiolase and therefore β-oxidation (Abo Alrob and Lopaschuk, 2014). Lactate has significant effects on energy substrate partitioning via mass action, cell redox control, allosteric binding, and chromatin reprogramming via histone lactylation (Brooks, 2020). Further, the injection of ketone bodies (KBs) decreases lactate formation and glycolysis (Cox et al., 2016). Like the treatment in PDH complex deficiency child, whether ketogenic diets or supplementation with KBs will improve fat oxidation and decrease lactate production in septic subjects requires further investigation. In addition, KBs can prevent oxidative stress and the NLRP3 inflammasome in inflammatory illness models (Shimazu et al., 2013), and subcutaneous KBs delivery guards against peripheral and neuroinflammation in sepsis (Ryan and O’Neill, 2020).

## 4 The influence of lactylation-related signaling pathways on macrophage polarization in sepsis

During sepsis, under the combined influence of various factors, such as pathogens, tissue damage, an inflammatory microenvironment, and metabolic products, macrophages promote a new balance between tolerance and resistance mechanisms in the body through metabolic reprogramming (Martins et al., 2019; Willmann and Moita, 2024).

Since its discovery in 2019 as a novel epigenetic modification at lysine residues, histone lysine lactylation (Kla) has gained recognition for its crucial function in connecting the cellular metabolite lactate to epigenetic regulation. Histone Kla is biochemically attached to lysine residues through the incorporation of a lactyl group (Zhang et al., 2019). The majority of the discovered histone Kla sites, H3K18la, H3K27la, H3K14la, H3K9la, H3K56la, and H4K12la, are found on H3 and H4 (Wang et al., 2024). Like other acylation modifications, histone Kla activates gene expression and is involved in cellular metabolism, inflammatory responses, and embryonic development. Zhang et al. revealed that LPS-mediated M1 macrophages elevated lactate levels by glycolysis and increased histone lactylation, increasing the levels of Arg1 and other wound healing-related genes, suggesting a phenotypic shift to M2 immunosuppressive macrophages (Zhang et al., 2019; Yang et al., 2020), promotes the tolerance mechanism to pathogens in diseases. They identified a modulatory mechanism in which both endogenous and exogenous lactate, resulting from aerobic glycolysis and MCT in M1 macrophages, respectively, were converted to lactoyl coenzyme A, which subsequently attaches a lactoyl moiety to histone lysine tails in the presence of p53 and acetyltransferase p300 (Zhang et al., 2019; Varner et al., 2020; Ma et al., 2022). Then, it was identified that aerobic glycolysis induces lactate accumulation in M1 macrophages, which results in pyruvate kinase M2 (PKM2)’s lactylation modification at K62, activating the tetrameric form of PKM2 and decreasing dimerization and nuclear translocation. This suppressed the macrophage Warburg effect, reduced lactate generation, and increased the M1 to M2 macrophage transition, which is called “the PKM2 paradox in the Warburg effect” (Dichtl et al., 2021; Wang J. et al., 2022). This pathway reduced the levels of M1 phenotypic genes in macrophages *via* the HIF-1α/PKM2 axis, promoting M2 features and mitigating the LPS-induced M1 phenotype (Palsson-McDermott et al., 2015; Wiese et al., 2021). Lactate enhances M2 macrophage polarization through HIF-1α signaling and induces histone lactylation, facilitating immune homeostasis (Zhang et al., 2019; Zhang and Li, 2020). In sepsis, it modulates the levels of inflammatory cytokines, increases the levels of Arg1, and induces macrophages’ anti-inflammatory function. Arg1 overexpression is associated with a macrophage-resolving phenotype (Chu et al., 2022; Chen et al., 2024). Ma XM et al. studied the pathogenesis of diabetic cardiomyopathy and indicated that although lactate can promote H3K12 and H4K18 lactylation in macrophages, it primarily directed the inflammatory response in macrophages and stimulated HIF-1α and inflammatory factors, including IL-1β, through H4K12 lactylation in conditions with high levels of free fatty acids (Ma et al., 2024). Lactyl-CoA is a key precursor in both histone and non-histone lactylation processes. The activity of CoA transferases that catalyze the synthesis of lactyl-CoA from lactate is strongly influenced by pH levels (Liao et al., 2025). Recent studies have identified alanyl-tRNA synthetases (AARS1/2) as unique enzymes that sense intracellular lactate and convert it into lactyl-CoA in an ATP-dependent manner to catalyze histone lactylation. This positioning makes lactylation a direct readout of metabolic changes, dynamically modifying lysine residues on histones to regulate gene expression during hypoxia or inflammation (Li et al., 2024). Lactylation is thus expected to influence the balance between tolerance and resistance in tissues by impacting macrophages’ metabolic reprogramming. Non-histone lactylation modification is also crucially involved in macrophage phenotype differentiation. Lactylation of lysine on histones exhibits slower (24 h) kinetics than does acetylation (6 h) in lung (Chen et al., 2021). Non-histone protein Kla affects protein levels and functions more directly by altering their stability, activity, or localization, whereas histone Kla affects gene accessibility and transcription (Zhang et al., 2025). To reestablish a new balance in Sepsis, histone and non-histone lactylation, along with other protein post-translational modifications, work together in metabolic reprogramming.

## 5 Role of other PTMs in sepsis-associated macrophage polarization

Among these post-translational modifications (PTMs), methylation, acetylation, and lactylation are important regulatory mechanisms governing macrophage polarization during sepsis. By comparing these common PTMs with lactylation, we can further clarify the role of lactylation in therapeutic strategies for sepsis.

### 5.1 Methylation and lactylation

Methylation refers to the transfer of a methyl group from S-adenosylmethionine (SAM) to lysine or arginine residues on proteins. This modification mainly occurs at specific sites on histones—H3K4, H3K9, H3K27, H3K36, and H3K79—with H3K9 being a notable competitive site for lactylation (Zhang and Li, 2020). Lactylation reflects the acute metabolic state of the cell, depends on lactyl-CoA, and can be reversed by deacetylases such as histone deacetylase (HDAC)1–3 and CobB (Chu et al., 2022). Methylation generally establishes stable and long-lasting epigenetic marks, whereas lactylation is highly dynamic and responsive to rapid metabolic fluctuations during inflammation (Wang J. et al., 2022).

Competition between methylation and lactylation at lysine residues (such as H3K9 or H3K18) forms a complex regulatory mechanism. This interplay allows lactylation to counteract repressive methyl marks during the resolution phase of inflammation, particularly as macrophages shift from a pro-inflammatory M1 phenotype to an anti-inflammatory M2 phenotype. Functionally, this metabolism-epigenetics crosstalk enables lactylation to modulate inflammatory responses. In macrophages, H3K18la promotes anti-inflammatory gene expression and may counterbalance the inhibitory effects of methylation on adjacent residues (Chen et al., 2024). In addition, lactate—the substrate for lactylation—can indirectly affect the availability of SAM, partly through mechanisms involving hypoxia-inducible factors, thereby influencing methylation patterns (Ma et al., 2024).

### 5.2 Acetylation, lactylation, and phosphorylation

The interaction between phosphorylation and lactylation is particularly important in metabolic regulation and sepsis. Phosphorylation indirectly affects lactylation by regulating glycolytic flux and lactate production. Phosphorylation of enzymes such as pyruvate kinase M2 (PKM2) and lactate dehydrogenase (LDH) promotes lactate accumulation, a hallmark of the Warburg effect (Wu et al., 2024). Elevated lactate levels subsequently drive histone lactylation, linking phosphorylation and lactylation dynamically. This mechanism highlights how PTMs are interconnected and co-regulated. Acetylation refers to the addition of an acetyl group to lysine residues on proteins, a process catalyzed by acetyltransferases (HATs/KATs) and reversed by histones deacetylases (Gil et al., 2017). It commonly occurs at multiple sites on histones, such as H3K9, K14, K18, and K23 (Arnaudo and Garcia, 2013), many of which are also frequent targets of lactylation.

Acetylation involves the addition of an acetyl group and is derived from acetyl-CoA, a common metabolite produced through OXPHOS of carbohydrates, lipids, and proteins. Lactylation, on the other hand, is mainly facilitated by lactate generated during aerobic glycolysis. Lactylation specifically adds lactyl groups derived from lactate, a byproduct of glycolysis, making it a direct indicator of cellular metabolic states, particularly under hypoxic or inflammatory conditions. This direct metabolic sensitivity positions lactylation as a key sensor of glycolytic flux. The accumulation of acetyl-CoA due to OXPHOS and acetylation, or the accumulation of lactate due to enhanced glycolysis and lactylation, has a strong regulatory effect on macrophage polarization. At the epigenetic intersection of lactylation and acetylation, the PDH complex catalyzes the irreversible oxidation of pyruvate generated by glycolysis to acetyl-CoA, priming the TCA cycle, initiating mitochondrial OXPHOS, and eventually driving ATP generation (Stacpoole and McCall, 2023). Several small compounds control PDH complex post-transcriptionally, primarily through reversible phosphorylation. Pyruvate dehydrogenase kinase (PDK) overexpression phosphorylates serine residues on the E1α subunit, rendering the complex inactive under anaerobic or inflammatory circumstances. This shifts carbohydrate metabolism away from OXPHOS and toward cytoplasmic pyruvate degradation to lactate, increasing glycolysis and facilitating the removal of pathogens. Two isoforms of pyruvate dehydrogenase phosphatase (PDP) 1 and 2dephosphorylate E1α and restore catalytic activity in aerobic environments. The PTM of histones may also be facilitated by acetyl-CoA, which is produced by glycolysis and other processes. The acetylation of histone lysine residues is a significant form of PTM-mediated epigenetic control. Gene transcription is made possible by the acetylation of conserved lysine residues on chromatin, which opens up the chromatin structure (Bannister and Kouzarides, 2011). In summary, acetylation neutralizes the positive charge on lysine residues, weakening electrostatic interactions between DNA and histones to create a more open chromatin structure amenable to transcription and providing a critical metabolic signal for cell growth and proliferation, promoted tolerance mechanisms and injury repair in sepsis (Lee et al., 2014). Lactylation can promote the shift of macrophages from the pro-inflammatory M1 phenotype to the anti-inflammatory M2 phenotype by enhancing anti-inflammatory responses, thereby increasing tolerance mechanisms during sepsis (Liu et al., 2019). When oxidative phosphorylation predominates, acetylation serves as the major form of protein modification, whereas lactylation becomes the dominant modification during anaerobic glycolysis. As interconnected yet functionally distinct regulators of cellular physiology, acetylation broadly fine-tunes cellular functions, while lactylation acts as a rapid and dynamic sensor of metabolic flux. However, the interactions between these two modifications remain unclear and require further investigation (**Table 1**).

**Table 1:** Major histone modifications and their pathophysiological effects in sepsis.

Histone modification	Mechanism	Target genes/pathways	Pathophysiological effect	Ref
H3K27me3	Catalyzed by EZH2; repressive mark	Sox9, IL-12 promoter	Suppresses gene expression; associated with immune cell dysfunction, AKI and reduced IL-12 in dendritic cells	(Li et al., 2023)
H3K4me3	Activating mark at gene promoters	NF-κB target genes	Enhances transcription of inflammatory genes; its loss linked to long-term immune suppression post-sepsis	(Davis et al., 2019)
H3K18la	Addition of lactyl group from lactate via p300	EGR1, RhoA	Promotes transcription of genes involved in repair and inflammation; associated with ALI, AKI, and immunosuppression	(Zhang et al., 2019; Chu et al., 2022; Qiao et al., 2024; Lu et al., 2025)
H3K27ac H3K4me1	Enhancers are involved in cytokine response and NF-κB signaling	NF-κB signaling,	LPS can induce the accumulation of histone H3K27ac and H3K4me1, but the accumulation of H3K27ac precedes that of H3K4me1.	(Novakovic et al., 2016)
Deacetylation	Removes acetyl groups; represses gene transcription	TNF, IL-1*β*	Promotes immunosuppression; HDAC1/2 modulates TLR responses and contributes to endotoxin tolerance	(Urdinguio et al., 2019)

EZH2, enhancer of zeste 2 polycomb repressive complex 2 subunit; Sox9, SRY-Box Transcription Factor 9; AKI, acute kidney injury; NF-κB, nuclear factor kappa B; HATs, histone acetyltransferases; Foxp3, forkhead box P3; EGR1, early growth response protein 1; ALI, acute lung injury; NET, neutrophil extracellular trap; TNF, tumor necrosis factor; HDAC1/2, histone deacetylase 1/2; TLR, toll-like receptor.

## 6 Therapeutic frontiers in the regulation of lactate and lactylation in sepsis

**Figure 2:**
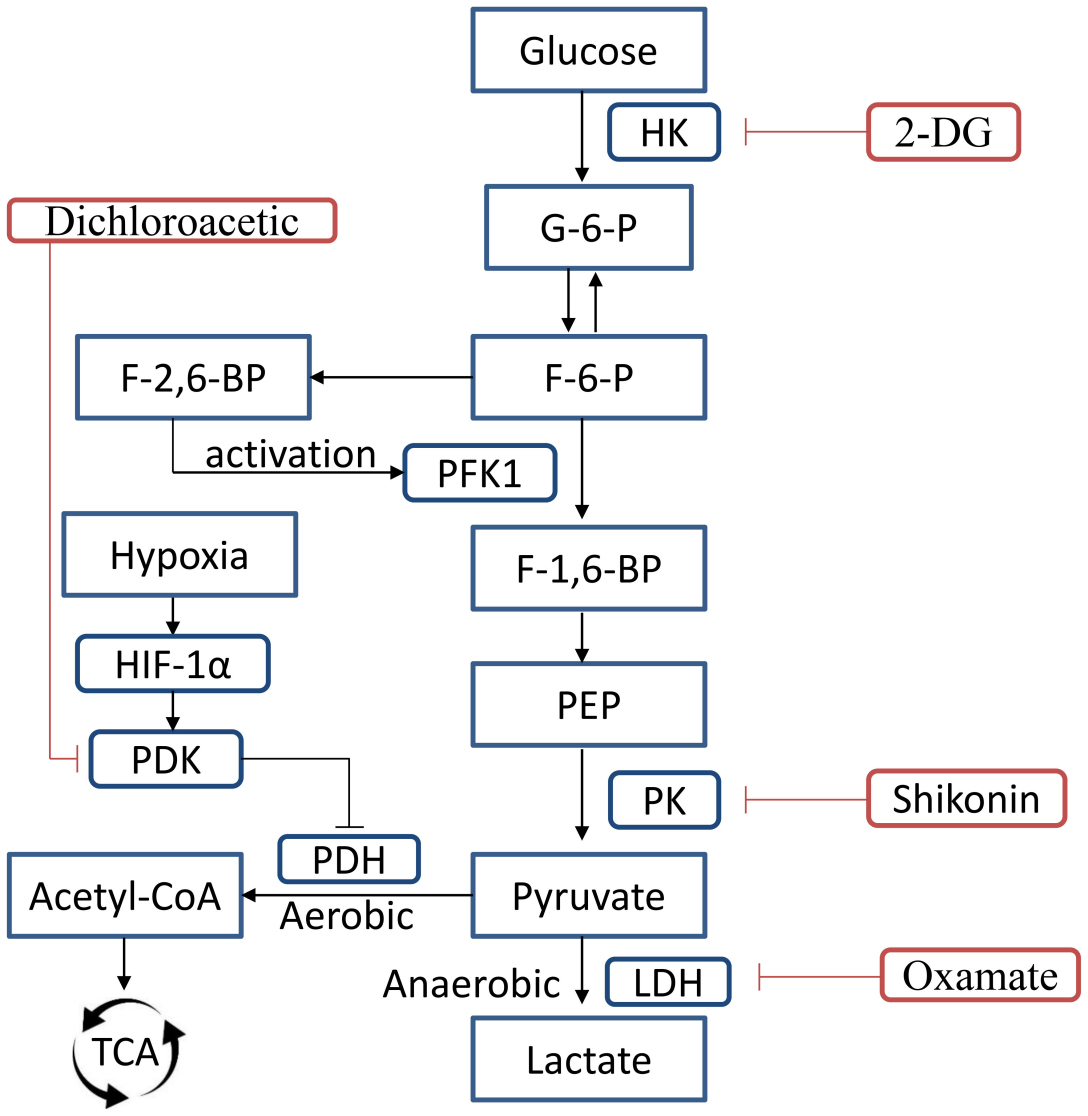
During glycolytic metabolism in macrophages, the first rate-limiting enzyme is HK, which catalyzes the conversion of glucose into G-6-P. The primary rate-limiting step of glycolysis is the PFK1-catalyzed conversion of F-6-P to F-1,6-BP. PFKFB catalyzes F-6-P transformation into F-2,6-BP, the most potent PFK1 activator. Another rate-limiting enzyme, PK, converts PEP to pyruvate. PKM2, an isoform of PK, promotes both macrophage inflammatory responses and aerobic glycolysis. PDK1 is an essential regulator of glucose metabolism that is responsible for the inactivation of PDH through the process of phosphorylation, hence inhibiting the conversion of pyruvate to acetyl-CoA and promoting lactate generation *via* glycolysis. HK2, hexokinase 2; PK, pyruvate kinase; PKM2, pyruvate kinase M2; LDH, lactate dehydrogenase; G-6-P, Glucose-6-phosphat; F-6-P, fructose 6-phosphate; F-1,6-P, 1,6-fructose diphosphate; PFK1, Phosphofructokinase-1; PEP, phosphoenolpyruvate; PDK, Pyruvate Dehydrogenase Kinase; PDH, pyruvate dehydrogenase; LDH, lactate dehydrogenase.

Histone Kla, MCT, and LDH are the primary targets in the control of lactate and lactylation (**Figure 2**). The last stage of glycolysis, which converts pyruvate to lactate, is catalyzed by LDH, which is widely distributed throughout the body’s tissues and organs. Irrespective of the increased rate of glycolysis and the decreased rate of selected substantial pyruvate-malate-dependent oxygen consumption, energy metabolism problems arise during Sepsis. Serum LDH levels are significantly elevated in sepsis patients due to the previously described reasons (Yu et al., 2024). LDH adopts a bilobal shape with two domains: a smaller domain devoted to the substrate pyruvate and a larger Rossmann domain that binds the cofactor NADH. Based on their mechanism of action, the LDH inhibitors identified to date can be divided into several categories. Among them are dual (substrate and cofactor) competitive inhibitors, cofactor (NADH) competitive inhibitors, and substrate (pyruvate) competitive inhibitors (Rai et al., 2017; Sharma et al., 2022). According to recent studies, oxamate, (R)-GNE-140, and fargesin are the medications that impact histone Kla. An inactive enzyme complex is created when oxamate and pyruvate compete for binding to LDH. It has been demonstrated that this association inhibits LDH activity, which in turn affects histone Kla levels, particularly H3K18la (Sun et al., 2023). This inhibition may improve the effectiveness of CAR-T-cell therapy. Histone Kla levels were considerably decreased by treatment with 10 μM (R)-GNE-140 (Dai et al., 2023). Similarly, fargesin’s potential as a therapeutic approach is demonstrated by its inhibition of LDH expression, thereby lowering H3Kla levels in non-small cell lung cancer (NSCLC) (Guo et al., 2024).

MCT1 and MCT4 are essential for improving lactate shuttling. The most powerful MCT inhibitors share comparable structural features. Among the inhibitors, the pyrrolidine derivative AZD3965 is distinguished by its strong inhibition of MCT1 and high affinity (Wang et al., 2021). In melanoma cells, AZD3965 enhances lactate levels and prevents lactate export (Tasdogan et al., 2020). Following treatment of mouse myoblast C2C12 cells with AZD3965, histone Kla levels are significantly increased (Dai et al., 2023).

Histone Kla is the target of the experimentally verified inhibitor C646 (Bowers et al., 2010). In myoblasts treated with lactate, this molecule has been shown to decrease H3K9la expression (Dai et al., 2023) substantially. However, C646’s effectiveness and specificity still need improvement. According to recent studies, elevated histone Kla accelerates disease progression, making eraser activators viable treatment options. Meanwhile, subsequent research may find that the disease is affected by lower levels of histone Kla, necessitating investigation of related inhibitors. Targeting lactylation-modifying enzymes, such as p300/CBP, histone deacetylases, and sirtuins (deacetylases), can alter the extent (Ciarlo et al., 2013; Moreno-Yruela et al., 2022). Controlling these enzymes can affect the lactylation balance, improving the inflammatory state and immunological response in sepsis patients. To clarify the upstream and downstream regulatory mechanisms of these lactylation enzymes, further research is needed.

2-Deoxy-D-glucose(2-DG), and oxamate (Maio et al., 2024), have demonstrated potential in mitigating systemic inflammation in sepsis models, underscoring their prospective therapeutic uses (Tan et al., 2020; Pan et al., 2022). In sepsis, these inhibitors reduce glycolysis rate, decreasing intracellular lactate, which suppresses the inflammatory reactions and tissue damage by inducing autophagy via the lactate/SIRT3/AMPK pathway (Zhong et al., 2019; Tan et al., 2021; Dai et al., 2022). Therefore, knocking down hypoxia-inducible factor 1α, pyruvate kinase isoenzymes M2, and Gly-related genes (Ding et al., 2021; Cai et al., 2022) can modulate lactate production via glycolytic pathways, and is a promising treatment approach for sepsis (Chen et al., 2021). Shikonin is a PKM2 inhibitor, which can reduce lactate generation, high mobility group box 1 protein (HMGB1) release, and septic mouse mortality in Adenosine 5’-monophosphate-activated protein kinase-deficient mice (Yang et al., 2014; Leite-Avalca et al., 2019).

## 7 Conclusion

Lactate is an essential energy intermediate found at the junction of oxidative and glycolytic metabolism. During sepsis, hypoxia and bacterial infection promoted anaerobic glycolysis, increased lactate levels and lactylation (Suetrong and Walley, 2016). Macrophages, as key regulators of immune responses with significant adaptability, undergo dynamic phenotypic alterations to accommodate changes in inflammatory and metabolic cues. Lactate restricts the over-inflammation of M1 macrophages through negative feedback by regulating histone or non-histone lactylation to promote the tolerance mechanisms in sepsis. Therefore, it was inferred that macrophage inhibition by targeting glycolysis, modulating lactylation, and identifying new lactylation sites are potential therapeutic avenues to improve sepsis prognosis. These approaches will provide novel perspectives and methods for treating sepsis by modulating immune cells’ metabolic state and inflammatory response. However, to ensure the clinical efficacy and safety of these treatment avenues, further validation is required. Moreover, lactyl-histones are considered potential bio-indices for sepsis prognosis and diagnosis (Bo et al., 2021). These strategies may become integral to sepsis treatment in the future, providing a wide range of therapeutic options for patients.
